# Three-Dimensional Monitoring of Plant Structural Parameters and Chlorophyll Distribution

**DOI:** 10.3390/s19020413

**Published:** 2019-01-20

**Authors:** Kenta Itakura, Itchoku Kamakura, Fumiki Hosoi

**Affiliations:** Graduate School, University of Tokyo, Tokyo 113-8657, Japan; itakura-kenta095@g.ecc.u-tokyo.ac.jp (K.I.); ik38@g.ecc.u-tokyo.ac.jp (I.K.)

**Keywords:** azimuthal angle, chlorophyll content, growth monitoring, image analysis, leaf inclination angle, plant, remote sensing, structural parameter, structure from motion, three-dimensional (3D) imaging

## Abstract

Image analysis is widely used for accurate and efficient plant monitoring. Plants have complex three-dimensional (3D) structures; hence, 3D image acquisition and analysis is useful for determining the status of plants. Here, 3D images of plants were reconstructed using a photogrammetric approach, called “structure from motion”. Chlorophyll content is an important parameter that determines the status of plants. Chlorophyll content was estimated from 3D images of plants with color information. To observe changes in the chlorophyll content and plant structure, a potted plant was kept for five days under a water stress condition and its 3D images were taken once a day. As a result, the normalized Red value and the chlorophyll content were correlated; a high R^2^ value (0.81) was obtained. The absolute error of the chlorophyll content estimation in cross-validation studies was 4.0 × 10^−2^ μg/mm^2^. At the same time, the structural parameters (i.e., the leaf inclination angle and the azimuthal angle) were calculated by simultaneously monitoring the changes in the plant’s status in terms of its chlorophyll content and structural parameters. By combining these parameters related to plant information in plant image analysis, early detection of plant stressors, such as water stress, becomes possible.

## 1. Introduction

Accurate monitoring of plant growth and structural parameters is important for crop yield estimation, species identification, determining vegetative growth, tracking illness, and monitoring insect infestation [1,2,3]. Two-dimensional (2D) imaging techniques have been utilized for obtaining the structural parameters of plants [4]. However, a 2D image is obtained by projecting the corresponding three-dimensional (3D) object in one direction; thus, because plants have complex 3D structures, it becomes difficult to accurately estimate their structural parameters. Thus, because 2D projections filter out potentially important information and fail to exploit the full potential of shape analysis, measurement of 3D plant architecture has become very important in plant biology and plant breeding [5,6,7,8]. Many efforts have been made to develop methods for 3D plant structural parameters estimation using 3D image acquisition methods; these methods use a 3D scanner (LIDAR; Light Detection and Ranging) and a photogrammetric approach called “structure from motion” (SfM). Using these methods, plant structural parameters, such as the leaf area index, leaf inclination angle, location, height, and volume, can be estimated [9,10,11,12,13,14,15,16,17,18,19,20,21,22,23,24,25,26,27,28]. The SfM methods are especially useful as they offer detailed 3D models of small plants with affordable cameras if the sample image is clearly taken. 

In addition to these structural parameters, estimating those parameters that are related to the plant physiology is also important. Chlorophyll content is one widely known example of such a physiologically relevant parameter. Chlorophyll is a basic component of plants and plays a vital role in the plant photosynthesis process. Its content is closely related to the photosynthetic capacity and the foliar nitrogen content [29,30]. Thus, chlorophyll is a good indicator of the vegetative growth status, plant damage, and plant aging. Furthermore, accurate determination of the plant chlorophyll content has important implications for crop growth monitoring, fertilizer regulation, herbicide application, the diagnostics of plant stress and activity, and yield assessment [31,32,33].

Previous studies have attempted to estimate the plant chlorophyll content from 2D images [34,35,36,37]. However, chlorophyll is usually 3D-distributed, to optimize the light acquisition for photosynthesis. Therefore, monitoring of the 3D distribution of chlorophyll is important. Furthermore, analysis that combines information about both the chlorophyll distribution and plant structure is likely to yield more detailed information about plants. 

Estimations of the plant structural parameters and physiological parameters are often performed independently; thus, the methodology for exploring the relationship between the plant structural and physiological properties has not yet been sufficiently developed. 

Previously, plant chlorophyll content has been measured using chemical analysis; however, this process is destructive, laborious, and time-consuming. Alternatively, the plant chlorophyll content can be estimated using a SPAD meter, which is a handy and lightweight sensor. Yet, using this method, the chlorophyll distribution along the entire leaf surface cannot be obtained and the spatial 3D distribution of chlorophyll cannot be obtained [33,38,39,40].

A previous study utilized a 3D scanner (LiDAR) to measure canopy structures. Subsequently, some vegetation indices related to the plant chlorophyll content were calculated using the reflectance information obtained from a simulated forest canopy [41]. Eitel et al. [42] estimated the plant chlorophyll content using LiDAR with green laser intensity. These authors utilized 3D imaging; thus, they were able to observe the spatial distribution of chlorophyll. However, LiDARs with green lasers are not widely available. Additionally, LiDARs are relatively expensive.

In contrast, 3D images can be reconstructed from multiple 2D images, using the SfM approach. It is possible to estimate the 3D distribution of chlorophyll from the color information. In this study, the plant chlorophyll content was first estimated using color information from 3D images that were reconstructed using the SfM methodology. Subsequently, the time series data of the chlorophyll content and structural parameters were observed and an integrated analysis of the plant 3D structure and chlorophyll distribution was performed.

## 2. Materials and Methods

### 2.1. Plant Material and Its 3D Reconstruction

For the experiments, eggplant plants (*Solanum melongena*) with height of ~40 cm and six leaves each were used. The experiment was conducted indoors and a lamp was positioned at a height of 80 cm. It was desired that the target leaves would be illuminated by the light. Firstly, to obtain the plant 3D images, 2D images were acquired from multiple views. The camera used for the acquisition of these images was a Canon EOS M2 (Canon Inc., Tokyo, Japan). The camera was handheld and moved around the sample in a circular fashion. Fifty images were recorded for one sample in order to obtain clear 3D models. The distance from the camera to the plant ranged from 30 cm to 100 cm. It is desirable to obtain more than 20 images. If the sample is not too large, 50 images will be enough to reconstruct a detailed 3D image. In the present study, the images were taken from an oblique angle. The resolution of the images was 3456 × 5184 pixels. Lower resolutions, such as 2000 × 2000 pixels, would also be acceptable. The resolution used should be determined based on the sample and environmental conditions. For the camera calibration and 3D point cloud image construction using the SfM approach [43,44,45], the software Agisoft Photoscan Professional (Agisoft LCC, Russia) was used. To assign a spatial scale and coordinates to the reconstructed 3D models, a cube-shaped box (13 cm × 12 cm × 9 cm) was positioned next to the plant [46]. 

### 2.2. Chlorophyll Content Estimation from Reconstructed 3D Images

**Figure 1:**
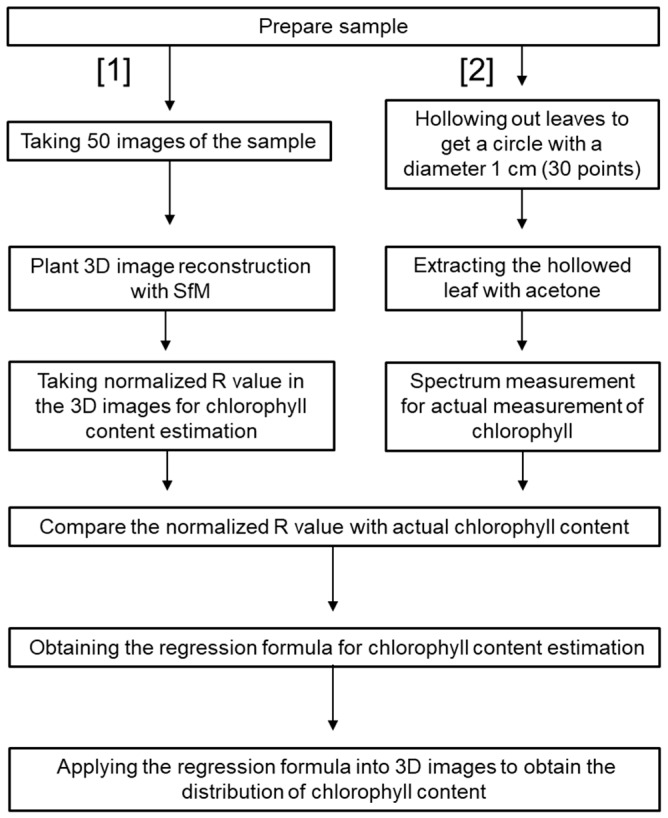
Flowchart of the validation of chlorophyll estimation from the plant 3D images.

The experimental workflow of the plant chlorophyll content estimation is shown in Figure 1. After the 3D reconstruction of the prepared sample, its chlorophyll content was estimated using the normalized Red value (R/(R+G+B)). Chlorophyll absorbs red light; hence, as the plant chlorophyll content increases, the reflectance decreases and a correlation between the plant chlorophyll content and the normalized Red value is observed. Other parameters related to plant physiological information, such as the plant carotenoid content, could potentially be estimated using color information and other invisible ranges. For this estimation, five potted eggplants were used. A total of 30 points were selected at random and the normalized Red value for each point was obtained. For the actual chlorophyll content measurement, the areas on the leaves corresponding to the selected points in the 3D images were hollowed using a hole punch. Next, the chlorophyll content in these areas was extracted using 80% acetone for 2 days. The amount of extracted chlorophyll was measured using a spectrometer (Jasco V570, Tokyo, Japan) and the actual chlorophyll content was obtained using the formula reported by Porra (1989) [47]. The relationship between the normalized Red value and the corresponding actual chlorophyll content value was calculated. Subsequently, a regression formula obtained from the relationship was applied to all of the points in the 3D models and the spatial distribution of the plant chlorophyll content was obtained. To evaluate the chlorophyll estimation accuracy, cross-validation was performed. 

### 2.3. Observations of the Alternation of Temporal and Spatial Plant Parameters

To monitor changes in the plant chlorophyll content and other plant structural parameters, the plant samples were kept in a dark room at 20 °C. The samples were put under a lamp once a day and their 3D images were obtained using the SfM method. After the 3D reconstruction, the chlorophyll content, leaf inclination angles, and azimuthal angles of the samples were calculated. By observing changes in the leaf inclination and azimuthal angles at different points of the leaves, structural changes in the different areas of the samples could be observed. During this experiment, water was not provided to the samples. To estimate the leaf inclination angle from the 3D images, a plane was fitted around a target point and its neighboring points [23]. The points used were the ones located within a cube whose centroid was the target point and the length of one side of the cube was 0.5 cm. Next, the zenith angle and the azimuthal angle of the vector normal to the fitted plane were calculated for all of the samples. The zenith angle corresponds to the leaf inclination angle.

## 3. Results and Discussion 

### 3.1. Estimation of Chlorophyll Content from 3D Images

**Figure 2:**
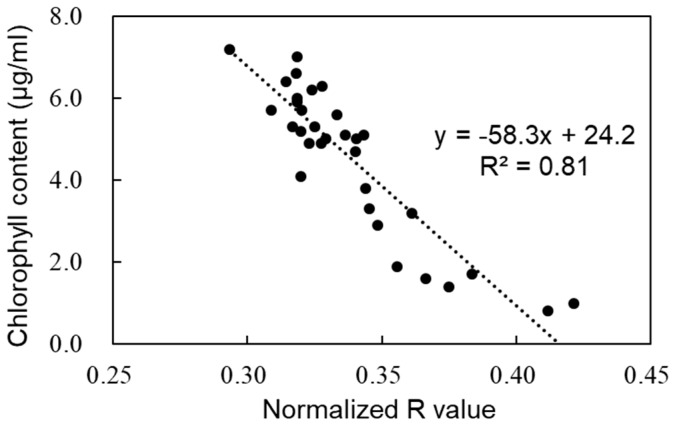
Relationship between the normalized Red value at different points in the plant 3D images and the local plant chlorophyll content.

**Figure 3:**
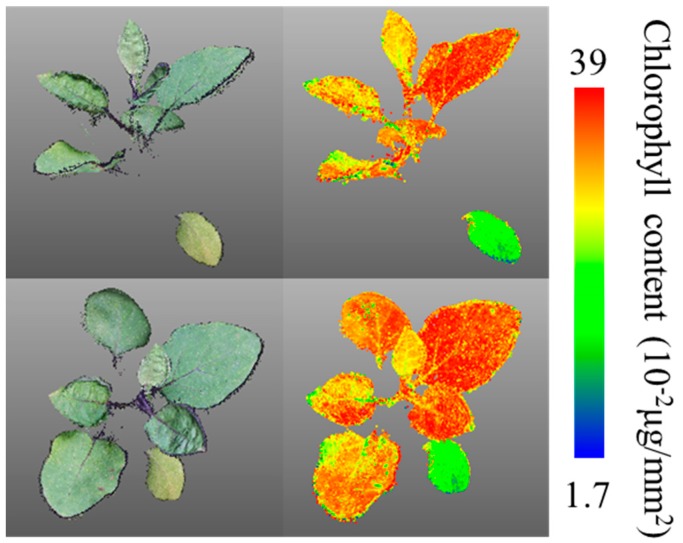
Reconstructed 3D images and distributions of chlorophyll content. Panels (**a**) and (**c**) show the same 3D images viewed from different angles. Panels (**b**) and (**d**) show the corresponding distributions of the chlorophyll content.

Figure 2 shows the relationship between the normalized Red value of the plant 3D images and the plant chlorophyll content (n = 30). The normalized Red value and the plant chlorophyll content were correlated, with a high R^2^ value (0.81). In the cross-validation study, the absolute error was 4.0 × 10^−2^ μg/mm^2^, implying that the plant chlorophyll content was estimated accurately. Figure 3 shows a reconstructed 3D image and the corresponding distribution of the chlorophyll content. Figure 3a,c shows the same 3D images viewed from different angles. Figure 3b,d shows the corresponding distributions of the chlorophyll content. The surrounded leaf in Figure 3b is larger and well-grown; accordingly, its chlorophyll content is higher. In contrast, the encircled leaf in Figure 3d is comparatively young and its chlorophyll content is comparatively low, because of the leaf immaturity. The leaf on the bottom right in Figure 3b,d is the oldest and its chlorophyll content is the lowest. As mentioned above, the chlorophyll content differs across leaves; in addition, the content also varies across individual leaves. Chlorophyll is heterogeneously distributed within a leaf [48,49]. Other 2D-based imaging methods (such as hyper-spectral imaging) also estimate the chlorophyll content distribution within leaves [50,51]. However, it is desirable to observe the spatial distribution of the plant chlorophyll content along with the 3D positional information. The method proposed in this study enables detailed monitoring of the plant chlorophyll content at different points in 3D images. Detailed 3D distributions of the plant chlorophyll content and other plant structural parameters can be measured and analyzed simultaneously, as shown later. The chlorophyll content estimation with green and blue value was also conducted, however, a good correlation could not be obtained. 

### 3.2. Time Series Observations of Chlorophyll Content and Structural Parameters within One Leaf

**Figure 4:**
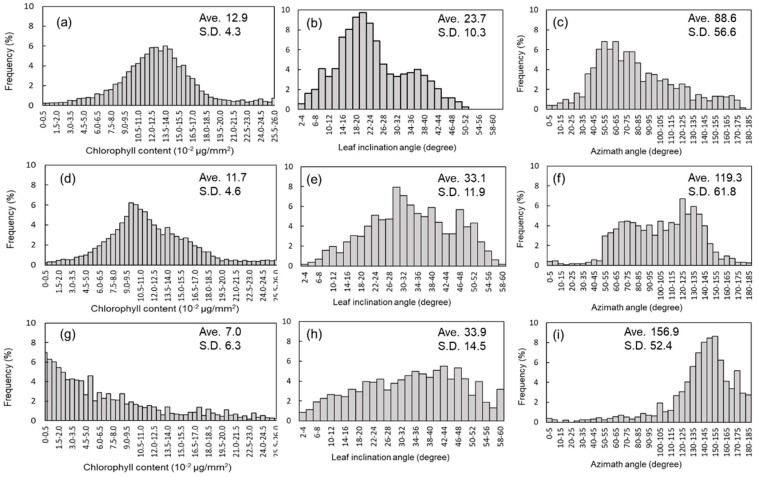
Examples of temporal snapshots of chlorophyll content, inclination angle, and azimuthal angle distributions within one leaf. Histograms (**a**–**c**), (**d**–**f**), and (**g**–**i**) capture the information for days 1, 3, and 5, respectively.

Figure 4 shows typical distributions of the plant chlorophyll content, leaf inclination angle, and azimuthal angle, for a sample leaf. The first, second, and third lines represent the profiles for day 1, day 3, and day 5, respectively. The values of chlorophyll content, leaf inclination angle and azimuthal angle among those days were significantly different (P < 0.05). This histogram was obtained from the first leaf from the bottom of the plant. From these histograms, it is clear that these parameters vary within a leaf. From day 1 to day 5, the chlorophyll content decreased; accordingly, the peak shifted to the left. The averages for day 1, day 3, and day 5 were 12.9, 11.7, and 7.0, respectively. In addition to the reduction in the chlorophyll content, the leaf inclination angle increased as the water stress on the leaves increased, as shown in the histograms in panels (b), (e), and (h) of Figure 4. The average values are 23.7°, 33.1°, and 33.9°, respectively. At that time, after being subjected to water stress, the leaves inclined with twisting, resulting in a change of the azimuthal angle. This is indicated by the fact that the peaks of the histograms in panels (c), (f), and (i) of Figure 4 shifted to the right. The average values were 88.6°, 119.3°, and 156.9º, respectively. For determining the leaf status (e.g., its chlorophyll content), detailed analysis was possible using these histograms, rather than using only averages. 

### 3.3. Time Series Data of Physiological and Structural Parameters from 3D Images

**Figure 5:**
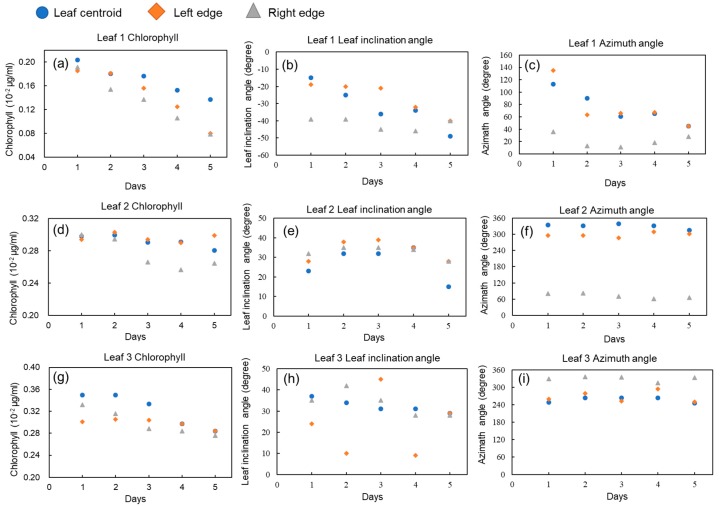
Time series data of chlorophyll content, inclination angle, and azimuthal angle, at a small point (leaf centroid, left edge, and right edge) within one leaf. The profiles of three leaves are illustrated. Panels (**a**–**c**), (**d**–**f**), and (**g**–**i**) capture the information for leaves 1, 2, and 3, respectively. Circles, squares, and triangles show the results for the leaves’ centroids, left edges, and right edges, respectively.

Figure 5 shows the time series data for the chlorophyll content, leaf inclination angle, and azimuthal angle for three leaves in one sample. Each figure includes data for different parts (leaf centroid, right edge, and left edge). Leaves 1, 2, and 3 correspond to old-age, middle-age, and young leaves, respectively. Their location in the sample was at the bottom, in the middle, and at the top, respectively. 

As shown in Figure 5a, the chlorophyll content of leaf 1, the oldest leaf, decreased strongly during the five observation days. In contrast, the rate of the chlorophyll content decrease was smaller for Leaves 2 and 3 (Figure 5b,c), which were also younger than leaf 1. For leaf 2 (Figure 5d), the chlorophyll content captured by the right edge (triangles) decreased compared with the leaf centroid and left edge, on days 2 and 3. The alterations of physiological parameters were different for different parts. As leaves get older, the role they play and their relative location in the plant change [52]. The level of chlorophyll increases in young expanding leaves, reaches the highest value when the leaves are mature, and then decreases substantially during senescence [53]. In previous studies of eggplant plants, chlorophyll content increased with leaf growth, was maximal at the 8- to 10-leaf stage, and then decreased [54]. In individual plants, the lower leaves are the first to senesce [55] and chlorophyll, protein, and RNA are moved and recycled in other developing sinks, such as new leaves [56]. In this study, water was not provided to samples and they suffered from water stress. As a result, stomatal closure and decreased photosynthetic functions were observed on the leaves as water stress developed [57]. When plant resources are limited, leaf senescence is important for increasing the dry matter production [58]. Thus, it is suggested that water stress accelerates the physiological and structural changes in the stressed plants. Using the current 3D imaging method, physiological and structural changes reflecting those biological phenomena can be monitored. 

For leaf 1 (the oldest leaf), the absolute value of the leaf inclination angle increased as the leaf weakened, as is shown in Figure 5b. Leaf 1 inclined downwards, whereas leaves 2 and 3 inclined upwards. To distinguish the difference, the value for leaf 1 was represented as a negative number. For leaf 1, the inclination angle was different across the different points (leaf centroid, right edge, and left edge), although those values increased similarly. For leaves 2 and 3, the values of these angles fluctuated, as is illustrated in Figure 5e,h. As for the azimuthal angle, leaf 1 inclined with twisting as it was subjected to water stress, so that its azimuthal angle changed significantly (Figure 5c). In contrast, leaves 2 and 3 shown in Figure 5f,i inclined in the vertical direction. Consequently, the distribution of the azimuthal angle did not change considerably. As for leaf 2, the leaf was folded at the center and the azimuthal angle varied strongly, even within the same leaf.

As mentioned above, the rates and/or the starting points of changes in the chlorophyll content, leaf inclination angle, and azimuthal angle differed across the leaves. In addition, these parameters varied across the area of individual leaves.

This method enables the observation of changes in the structural and physiological parameters in different areas of individual leaves. The leaf shapes are different in the small areas in the leaves and, in many cases, these variations can be related to stress. For example, citrus leaves are curved when the plant is subjected to water stress. This change can be tracked by observing changes in the azimuthal angles of leaves in different areas. Therefore, taking these small-area changes into consideration, early detection of plant stressors, such as water stress, becomes possible. Using this method, changes in the chlorophyll content and structural parameters can be tracked at the same time and it becomes possible to collect different data indicative of plant stress. This could help us with determining the structural and physiological properties of a target sample and it could be widely used, for example, in phenotyping. Moreover, by connecting the plant physiological and structural parameters, deeper knowledge about plant traits can be generated.

## 4. Conclusions

In this study, 3D images of eggplants were obtained using the SfM method. Chlorophyll content in small areas of leaves was estimated from color information of the 3D plant images and the obtained values were compared with actual values. The normalized Red value and the chlorophyll content were correlated; a high R^2^ value (0.81) was obtained. To better generalize this method for its application, more leaves should be examined and the same experiment should be repeated in the future. With additional plots, a more reliable calibration curve could be created and this would lead to its application. At the same time, this method allows the estimation of structural parameters, due to the 3D image acquisition. To observe changes in the chlorophyll content and plant structure, a potted eggplant was kept for five days under water stress and its 3D images were taken once a day. The chlorophyll content in the oldest leaf of the sample decreased and its leaf inclination angle increased as the leaf became weaker. The azimuthal angle also changed because it inclined with twisting. In addition, the chlorophyll content and structural parameters (leaf inclination angle and azimuthal angle) were different not only across other leaves, but also within individual leaves. The method used in the current study allows a better understanding of plant traits and improved plant monitoring, based on essential plant traits. In the future, plants other than eggplants should be examined. Furthermore, other parameters related to plant physiological information, such as the plant carotenoid content, should be estimated using color information and other invisible ranges.
